# Impact of Long-Term Plasma Storage on Cell-Free DNA Epigenetic Biomarker Studies

**DOI:** 10.3390/biom15070927

**Published:** 2025-06-25

**Authors:** Jianming Shao, Thao Nguyen, Zejuan Li

**Affiliations:** 1 Department of Pathology and Genomic Medicine, Houston Methodist Hospital, Houston, TX 77030, USA; 2 Houston Methodist Research Institute, Houston, TX 77030, USA; 3 Weill Cornell Medical College, New York, NY 10065, USA

**Keywords:** cell-free DNA, genomic DNA, plasma storage, 5-hydroxymethylcytosine, cancer

## Abstract

Impact of long-term plasma storage on biomarker analysis is critical for ensuring data reliability. Cell-free DNA (cfDNA) epigenetic markers, including 5-hydroxymethylcytosine (5hmC), have emerged for disease detection, prognosis, and treatment response. However, the effects of prolonged storage on 5hmC analysis remain unclear. We evaluated the quantity and quality of cfDNA and 5hmC sequencing analyses in 1070 plasma samples stored for up to 14 years from patients with solid tumors and acute myeloid leukemia (AML) and non-cancer individuals. In long-term stored plasma samples, cfDNA yield remained largely stable; however, uniquely mapped reads (UMRs) from 5hmC sequencing were significantly reduced in solid tumor and control samples. Notably, prolonged plasma storage independently contributed to increased genomic DNA (gDNA) contamination in solid tumor and AML samples and significantly correlated with decreased UMRs in control samples. Across all groups, samples with gDNA contamination exhibited significantly reduced UMRs. Furthermore, gDNA contamination independently compromised cfDNA fragment integrity, decreased sequencing library success in solid tumors, and reduced 5hmC sequencing UMRs across all groups. Therefore, extended plasma storage contributes to increased gDNA contamination, compromising cfDNA and 5hmC sequencing quality. Implementing measures to minimize gDNA contamination in long-term plasma storage is crucial for improving downstream cfDNA analysis reliability.

## 1. Introduction

Long-term stored plasma samples are valuable resources for discovering genetic and epigenetic cell-free DNA (cfDNA) biomarkers in retrospective studies and clinical trials [1,2]. cfDNA consists of DNA fragments originating from normal or malignant cells [3]. Compared with tissue biopsy, cfDNA analysis is minimally invasive, inexpensive, and reflects the temporal and spatial heterogeneity of malignant cells in cancer patients [4]. Genetic mutations and epigenetic markers in plasma cfDNA offer great potential for early diagnosis, disease prognosis, dynamic disease monitoring, and treatment guidance in cancer and other diseases [4]. To prevent cfDNA degradation, plasma is commonly isolated and stored at −80 °C until DNA extraction [5]. As cfDNA is present at very low concentrations, long-term storage may cause fragmentation and reduced cfDNA yield [6,7,8,9]. Despite prolonged plasma storage, no adverse effects on circulating tumor DNA mutation analysis have been observed [9]. However, the impact of prolonged plasma storage on cfDNA analysis of epigenetic markers is yet to be studied.

5-hydroxymethylcytosine (5hmC) is an emerging epigenetic marker with significant potential in disease detection, prognosis, and predicting therapeutic responses [2,10,11,12,13]. 5hmC is a cytosine modification resulting from oxidation by ten-eleven translocation enzymes [13]. 5hmC is most prevalent in gene bodies and enhancers, strongly correlating with gene expression. It offers advantages over RNA and genetic biomarkers due to its greater stability and abundance in bodily fluids [14]. Technologies such as nano-hmC-Seal have been developed to map and monitor 5hmC, enabling its use as a biomarker in cfDNA analysis.

5hmC-based signatures in plasma cfDNA have demonstrated efficacy as detection and prognosis markers for colorectal, gastric, lung, pancreatic, prostate, ovarian, nasopharyngeal, and esophageal cancer, lymphoma, acute myeloid leukemia (AML), and other malignancies [2,10,11,12,13,15,16,17,18,19,20]. Additionally, plasma cfDNA 5hmC has utility in minimal residue disease detection in AML [21] and predicting responses to immune checkpoint inhibitor therapy in lung cancer [22] and chemotherapy in ovarian cancer [23]. Because 5hmC exhibits tissue-specific signatures, it can indicate cancer origin [13,19,24]. Moreover, plasma cfDNA 5hmC can detect other conditions, such as type 2 diabetes, diabetic nephropathy, diabetic kidney disease, coronary artery disease, COVID-19 progression and myocardial injury, late-onset Alzheimer’s disease, and systemic lupus erythematosus [25,26,27,28,29,30,31]. As most studies rely on long-term stored plasma samples, understanding how storage impacts cfDNA 5hmC analysis is crucial for ensuring data reliability.

To assess the effects of plasma storage duration on the quality of cfDNA and 5hmC analysis, we evaluated 1070 plasma samples stored at −80 °C for up to 14 years from individuals with and without cancer. Considering factors such as age, sex, plasma storage time, cancer stage, and time between blood collection and plasma processing (elapsed processing time), we investigated cfDNA yield, cfDNA fragmentation, and genomic DNA (gDNA) contamination during cfDNA 5hmC analysis using nano-hmC-Seal coupled with next-generation sequencing (NGS, nano-hmC-Seal-Seq). We identified key factors to consider when analyzing long-term stored plasma samples and provide practical guidance for optimizing their use in both research and clinical practice.

## 2. Materials and Methods

### 2.1. Sample Collection

In this retrospective study, we analyzed 1070 peripheral blood samples collected between 2006 and 2022 at Houston Methodist Hospital (HMH). The samples included 211 non-cancer individuals, 622 patients with solid tumors (bladder, breast, colorectal, kidney, lung, ovarian, prostate, and uterine cancers), and 237 patients with AML (Table 1). Blood was collected into K_2_EDTA tubes. Plasma was isolated by centrifuging at 1350× *g* for 10 min at 4 °C and stored at −80 °C at the HMH Biorepository until analysis. Blood samples were processed immediately or after delays of up to 36 days. This study was approved by the institutional review board at HMH.

### 2.2. cfDNA Extraction

Plasma samples were further centrifuged twice at 13,500× *g* for 10 min at 4 °C. The final supernatant was transferred to a new microcentrifuge tube for cfDNA extraction. cfDNA was extracted from 0.11 to 1.4 mL plasma samples using a QIAamp circulating nucleic acid extraction kit and QIAvac system (Qiagen, Hilden, Germany) according to the manufacturer’s instructions.

### 2.3. cfDNA Quality and Integrity Assessment

We quantified cfDNA using the Qubit Fluorometer with dsDNA HS Assay Kit (Thermo Fisher Scientific, Waltham, MA, USA) according to the manufacturer’s instructions. cfDNA yield was measured as the DNA quantity per milliliter of plasma. As DNA capillary electrophoresis allows us to estimate DNA fragment integrity, we analyzed the integrity of cfDNA samples using the Agilent 2100 Bioanalyzer with the Agilent High Sensitivity Assay Kit (Agilent Technologies, Santa Clara, CA, USA). We defined fragment sizes of ~160 bp or a trimodal distribution of DNA sizes (~160, 320, and 480 bp) as cfDNA fragments, and fragments larger than 1000 bp as gDNA.

### 2.4. cfDNA 5hmC Analysis by Nano-hmC-Seal-Seq Assay

The nano-hmC-Seal-Seq assay was performed as previously described [17]. Briefly, cfDNA fragments containing 5hmC were first captured by the selective 5hmC chemical labeling strategy and then amplified by PCR. Paired-end sequencing (300 cycles) was performed on the NextSeq 550 or NovaSeq 600 instrument (Illumina, San Diego, CA, USA). We evaluated the quality of raw reads and trimmed adaptors and low-quality reads using Trimmomatic [32]. High-quality reads were mapped to the reference genome (GRCh37) using bowtie2 in end-to-end mode [33]. We removed PCR duplicates using SAMtools [34]. Uniquely mapped reads (UMRs) were used for cfDNA 5hmC sequencing quality assessment.

### 2.5. Statistical Analyses

cfDNA and UMR data were not normally distributed (Shapiro–Wilk test, *p* value < 2.2 × 10^−16^ and *p* value = 5.2 × 10^−15^, respectively), even after Box–Cox transformation. Therefore, the Kruskal–Wallis test and Wilcoxon Rank Sum test were used for comparison of cfDNA yield and UMRs across different variables. We performed univariable and multivariable quantile regression analyses to evaluate the impact of age, sex, plasma storage time, gDNA contamination, cancer stage, and processing delay on cfDNA yield and UMRs. For cfDNA yield and UMRs, univariable quantile regression analysis was performed at the 50th quantile and multivariable quantile regression analysis was performed at the 25th, 50th, and 75th quantiles. Quantile regression analyses were carried out using the quantreg package (version 6.1), and confidence intervals were estimated via bootstrapping using the boot package (version 1.3-31). We also performed univariable and multivariable logistic regression for cfDNA fragment presence, gDNA contamination, and 5hmC library preparation success. For all statistical tests, a *p* value < 0.05 was considered significant. All statistical analyses and visualizations were performed in R (version 4.5.1; R Core Team, 2024). Data manipulation was conducted using the dplyr package (version 1.1.4), and figures were generated with the ggplot2 package (version 3.5.2).

## 3. Results

### 3.1. cfDNA Yield in Long-Term Stored Plasma Samples

**Figure 1:**
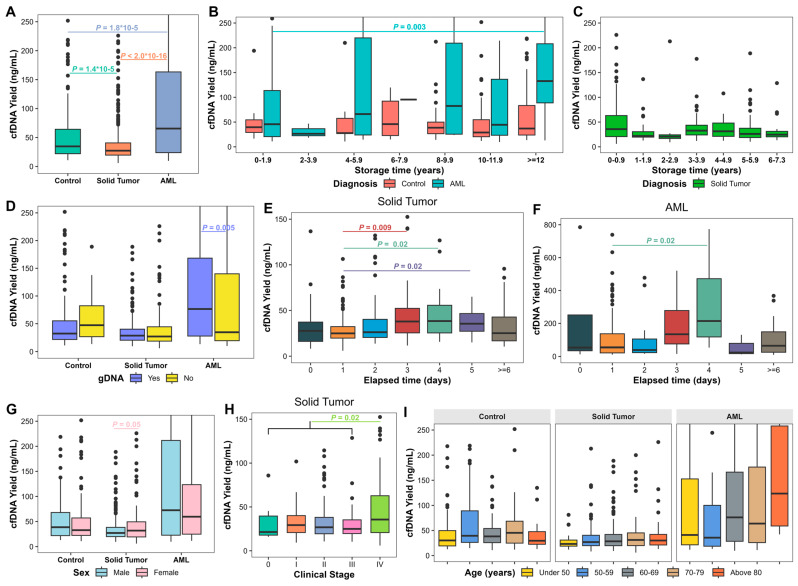
Cell-free DNA (cfDNA) yield in long-term stored plasma samples. (**A**) cfDNA yield in plasma samples from patients with solid tumors and acute myeloid leukemia (AML) and non-cancer controls. (**B**) cfDNA yield in plasma samples from patients with AML and controls based on plasma storage time. (**C**) cfDNA yield in plasma samples from patients with solid tumors based on plasma storage time. (**D**) cfDNA yield of plasma samples based on genomic DNA (gDNA) contamination. (**E**) cfDNA yield of plasma samples from patients with solid tumors based on elapsed processing time. (**F**) cfDNA yield of plasma samples from patients with AML based on elapsed processing time. (**G**) cfDNA yield of plasma samples from patients with solid tumors and AML and controls based on sex. (**H**) cfDNA yield of plasma samples based on age. (**I**) cfDNA yield of plasma samples from patients with solid tumors based on cancer stages. Kruskal–Wallis test or Wilcoxon Rank Sum test was performed. Bounds of box represent 25th and 75th percentiles and whiskers are Tukey whiskers. *p* values < 0.05 are indicated.

We first evaluated cfDNA yield in 1070 plasma samples. The median cfDNA concentration was 0.55 ng/μL (range, 0.16–25.50 ng/μL) with plasma samples having a median yield of 30.36 ng/mL (range, 5.98–4311.59 ng/mL). Plasma cfDNA yield in AML patients (median, 65.49 ng/mL; range, 9.67–4311.59 ng/mL) was significantly higher than in solid tumor patients (median, 27.28 ng/mL; range, 5.98–792.21 ng/mL; *p* < 2.0 × 10^−16^) and controls (median, 34.69 ng/mL; range 10.83–839.04 ng/mL; *p* = 1.8 × 10^−5^; Figure 1A). cfDNA yield from solid tumors was significantly lower compared to controls (*p* = 1.4 × 10^−5^; Figure 1A). Due to these differences, we assessed solid tumors, AML, and controls independently.

We further analyzed 414 samples from solid tumors and 220 from AML patients with known elapsed processing times, and all controls. cfDNA yield was not significantly affected by plasma storage duration in samples from AML, solid tumors, or controls, except in AML samples stored longer than 12 years (*p* = 0.003; Figure 1B,C). Plasma samples with gDNA contamination demonstrated a significantly higher cfDNA yield compared to samples without gDNA contamination in AML (*p* = 0.005; Figure 1D). Increased elapsed processing time resulted in significant increases in cfDNA yield on day 3 (*p* = 0.009), day 4 (*p* = 0.02), and day 5 (*p* = 0.02) in solid tumors (Figure 1E) and day 4 in AML (*p* = 0.02; Figure 1F) compared to day 1. Solid tumor samples from female patients showed higher cfDNA yields compared to males (*p* = 0.05; Figure 1G). cfDNA yield of Stage IV patients (median, 35.63 ng/mL; range, 5.98–333.18 ng/mL) was significantly higher compared to stages 0-III (median, 27.14 ng/mL; range, 9.30–548.57 ng/mL; *p* = 0.02; Figure 1H). cfDNA yield did not significantly vary with age (Figure 1I).

### 3.2. Factors Associated with Plasma cfDNA Yield

To evaluate factors that may have an impact on plasma cfDNA yield, we first performed univariable quantile regression analysis at the 50th percentile of cfDNA yield considering age, sex, plasma storage time, gDNA contamination, elapsed processing time, and cancer clinical stage. Increased plasma storage time (*p* = 0.001) and gDNA contamination (*p* = 0.02) were significantly correlated with increased cfDNA yield in samples from AML. Other factors did not show significant correlation with cfDNA yield.

To determine independent factors for cfDNA yield, we performed multivariable quantile regression analysis on cfDNA yield from 414 plasma samples with solid tumors, 220 with AML, and control samples. At the 50th quantile for AML samples, longer plasma storage time (*p* = 0.008) and older age (*p* = 0.02) were significantly associated with increased cfDNA yield (Table 2, Figure S1A,B). At the 25th quantile, gDNA contamination was significantly correlated with increased cfDNA yield in AML (*p* = 0.04) and approached significance in solid tumors (*p* = 0.07; Table 2; Figure S1C). All other variables did not demonstrate a significant impact on cfDNA yield.

### 3.3. Factors Associated with cfDNA Fragment Presence

DNA fragment integrity analysis demonstrated that cfDNA fragments were present in 893 (83.5%) of the 1070 plasma samples (Figure S2). We performed univariable logistic regression analysis to investigate factors that may affect the presence of cfDNA fragments. Prolonged plasma storage (*p* = 9.4 × 10^−4^), gDNA contamination (*p* = 9.5 × 10^−6^), and elapsed processing time (*p* = 0.03) were significantly correlated with reduced cfDNA fragment presence in solid tumors. Increased age in solid tumors (*p* = 6.6 × 10^−5^) and controls (*p* = 0.05) and cancer stage III (*p* = 0.01) and IV (*p* = 0.002) in solid tumors were significantly correlated with increased cfDNA fragment presence. Multivariable logistic regression analysis confirmed gDNA contamination as a negative independent factor for cfDNA fragment presence in solid tumors (*p* = 0.01; Table 3).

### 3.4. Factors Associated with gDNA Contamination

Among the 1070 cfDNA samples, 635 exhibited gDNA (339 in solid tumors, 141 in AML, and 155 in controls; Figure S3). To explore factors that contribute gDNA contamination in plasma cfDNA, we performed multivariable logistic regression analysis considering age, sex, plasma storage time, cancer types, and elapsed processing time (Table 3). Prolonged plasma storage time was significantly correlated with increased gDNA contamination in solid tumors (*p* = 4.9 × 10^−4^) and AML (*p* = 0.05; Table 3). We observed a significant correlation between increased processing elapsed time and gDNA contamination in solid tumors (*p* = 0.004; Table 3). Blood samples processed after one day demonstrated significantly increased gDNA contamination relative to same-day processed samples in solid tumors (Table S1). Samples from female patients showed negative correlation with gDNA contamination in both solid tumors (*p* = 0.007) and controls (*p* = 0.05; Table 3). Increased age also demonstrated a significantly negative correlation with gDNA contamination in solid tumors (*p* = 0.02; Table 3).

### 3.5. Factors Associated with Successful DNA Library Preparation for 5hmc Analysis

To perform 5hmC sequencing, we prepared a DNA library using the nano-hmC-Seal method. Among 893 cfDNA samples with cfDNA fragments, 745 samples (83.6%) had sufficient library DNA quantity and quality for NGS (Figure S4). We performed multivariable logistic regression analysis on samples from solid tumors (n = 335) and AML (n = 195) with elapsed processing time information and 180 control samples (Table 3). High cfDNA yield significantly correlated with successful library preparation in solid tumors (*p* = 0.004), AML (*p* = 4.1 × 10^−4^), and controls (*p* = 0.02; Table 3). In solid tumors, gDNA contamination was a significant negative factor for DNA library success (*p* = 0.008) while female sex was a positive independent factor for DNA library success (*p* = 8.6 × 10^−5^; Table 3).

### 3.6. Factors Associated with 5hmC Sequencing Quality

**Figure 2:**
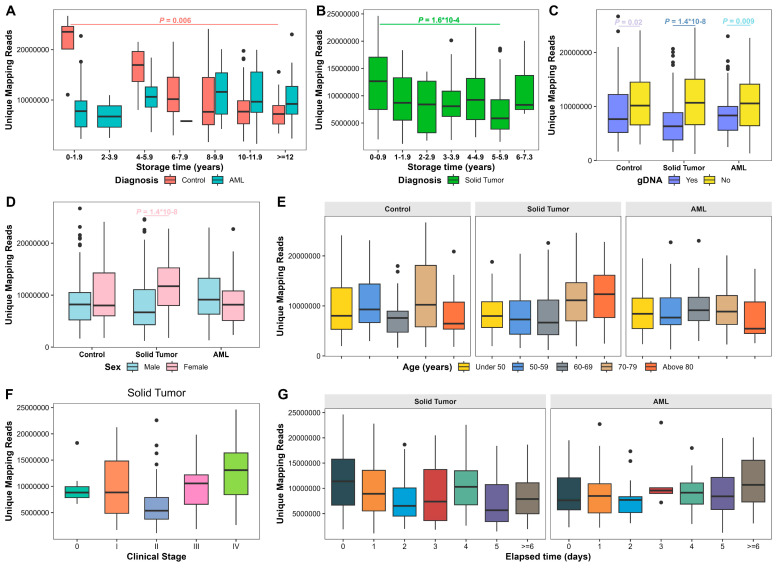
Uniquely mapped reads (UMRs) in 5hmC sequencing analysis. (**A**) UMRs of plasma samples from patients with AML and controls based on plasma storage time. (**B**). UMRs of plasma samples from patients with solid tumors based on plasma storage time. (**C**). UMRs of plasma samples based on gDNA contamination. (**D**). UMRs of plasma samples based on sex. (**E**) UMRs of plasma samples based on age. (**F**). UMRs of plasma samples from patients with solid tumors based on cancer clinical stages. (**G**) UMR yield of plasma samples from patients with solid tumors and AML based on elapsed processing time. Kruskal–Wallis test or Wilcoxon Rank Sum test was performed. Bounds of box represent 25th and 75th percentiles and whiskers are Tukey whiskers. *p* values < 0.05 are indicated.

We performed NGS on 745 samples with sufficient quantity and quality of library DNA. As UMRs reflect sequencing quality, we analyzed UMRs based on age, sex, plasma storage time, gDNA contamination, cancer stage, cfDNA yield, and elapsed processing time in 271 samples from solid tumor patients and 163 samples from AML patients with elapsed time information and 153 control samples. Overall, the median UMR was 8,428,123 (range: 1,190,583–26,690,091). UMRs were significantly reduced in samples stored for 5–5.9 years compared to under 1 year in solid tumors (*p* = 1.6 × 10^−4^) and in samples stored over 12 years compared to under 2 years in controls (*p* = 0.006; Figure 2A,B). UMRs were also significantly reduced in plasma samples with gDNA contamination in solid tumors (*p* = 1.4 × 10^−8^), AML (*p* = 0.009), and controls (*p* = 0.02; Figure 2C). Females exhibited significantly higher UMRs in solid tumors (*p* = 1.4 × 10^−8^; Figure 2D). UMRs did not demonstrate significant differences with increased age across all groups (Figure 2E), in late-stage cancer in solid tumors (Figure 2F), or with increased elapsed processing time in solid tumors and AML (Figure 2G).

To assess independent factors for UMRs, we performed multivariable quantile regression analysis. Increased plasma storage time was significantly correlated with decreased UMRs at the 25th quantile (*p* = 0.003), 50th quantile (*p* = 1.5 × 10^−9^), and 75th quantile (*p* = 5.6 × 10^−10^) in controls, and increased UMRs at the 25th quantile in AML (*p* = 0.02; Table 4; Figure S5A). gDNA contamination was significantly correlated with decreased UMRs at the 25th, 50th, and 75th quantiles in solid tumors (*p* = 0.008, 0.001, and 0.01, respectively), at the 50th and 75th quantiles in AML (*p* = 8.6 × 10^−4^ and 0.006, respectively), and 75th quantiles in controls (*p* = 0.04; Table 4; Figure S5B). High cfDNA yield was significantly associated with increased UMRs at the 25th quantile in AML (*p* = 0.01) and at the 25th (*p* = 0.004) and 50th (*p* = 0.03) quantiles in controls (Table 4; Figure S5C). Female sex was significantly associated with increased UMRs in solid tumors (*p* = 0.01 at the 25th quantile; *p* = 0.01 at the 50th quantile; *p* = 0.005 at the 75th quantile) but decreased UMRs at the 50th quantile in AML (*p* = 0.03; Table 4; Figure S5D).

## 4. Discussion

As long-term stored plasma samples are widely used for biomarker research, understanding the impact of prolonged storage is crucial for ensuring data reliability. To evaluate cfDNA quality in stored plasma, we analyzed cfDNA yield, fragment integrity, and gDNA contamination, and conducted genome-wide 5hmC profiling using NGS. Previous studies reported reduced cfDNA levels after prolonged plasma storage at −80 °C [6,7,8]. Our findings indicate that cfDNA yield remained stable in long-term stored plasma samples, except for samples stored beyond 12 years in AML. This suggests plasma storage can preserve cfDNA yield over extended periods. Additionally, prolonged storage independently increased cfDNA yield in a subset of AML samples (at the 50th quantile), potentially due to increased DNA release from plasma components, such as extracellular vesicles (EVs) [35] and platelets [36], during storage.

However, long-term storage of plasma samples may compromise downstream analysis. We found that extended plasma storage reduced 5hmC sequencing UMRs in solid tumors and controls and was an independent factor for decreased UMRs in controls. Prolonged plasma storage also independently increased gDNA contamination in solid tumors and AML. Moreover, gDNA contamination negatively impacted cfDNA fragment integrity and decreased sequencing library success in solid tumors, and reduced 5hmC sequencing UMRs across all groups. Therefore, the reduction in UMRs observed in prolonged stored samples may be attributed to increased gDNA contamination and/or cfDNA degradation over time [6,7,8,9]. gDNA contamination likely interferes with 5hmC analysis quality by reducing the proportion of cfDNA in the total DNA input.

We identified two primary sources of gDNA contamination. First, the time between blood collection and plasma processing contributes to gDNA release from blood cells [4,5]. Consistent with prior findings [5], blood samples processed more than one day after collection exhibited a significant increase in gDNA contamination. Second, prolonged plasma storage itself contributes to gDNA contamination. In our study, plasma samples were processed using low-speed centrifugation, which may leave EVs and platelets, which release gDNA during extended storage. To minimize contamination, additional plasma processing steps, such as a second high-speed centrifugation, may help remove DNA-containing components before storage. Therefore, rapid post-collection processing, additional centrifugation, and minimized plasma storage duration are recommended to improve cfDNA and 5hmC analysis quality.

Plasma samples from AML exhibited significantly higher cfDNA yield compared to solid tumors and controls, consistent with previous studies [37,38], reflecting the hematopoietic system as a primary cfDNA source [39]. In addition, delayed plasma processing time increased cfDNA yield, likely due to gDNA contamination. Higher cfDNA yield was significantly associated with successful 5hmC library preparation across solid tumors, AML, and controls, as well as increased UMRs in a subset of AML and control samples.

Plasma samples from solid tumor patients exhibited lower cfDNA yield compared to controls, which may be attributed to three factors. First, most solid tumor patients in our study were at early cancer stages, whereas previous studies have reported higher cfDNA yields in late-stage cancer patients compared to controls [4]. Second, the storage duration for solid tumor plasma samples (up to 7 years) was shorter than that of control samples (up to 13 years), and prolonged storage was significantly associated with increased cfDNA yield. Third, the majority (465) of the samples were collected post-surgical resection of the tumors. As the level of cfDNA is significantly correlated with tumor size [40], cfDNA level can be reduced after tumor removal.

We found that female sex was significantly associated with higher 5hmC sequencing library preparation success and increased UMRs in solid tumors. Previous research has reported sex-specific differences in 5hmC distribution [41], suggesting that the presence of an additional X chromosome in females may contribute to increased levels of 5hmC-enriched DNA fragments compared to males. However, further studies are needed to confirm this association. Overall, age did not have a significant effect on cfDNA yield, cfDNA fragment integrity, library preparation success, or UMRs across all groups.

Our study has several limitations. First, all samples were processed using the same method at a single institute, limiting comparisons with studies employing different plasma processing methods or collection tubes. Second, plasma sample storage times varied between solid tumor and control groups, making direct comparisons between the two challenging. Third, we used 5hmC profiling to assess sequencing quality, which may not fully represent findings from DNA methylation or genomic sequencing. Fourth, not all results were consistent across solid tumors, AML, and controls, possibly due to small sample sizes in certain groups, variable storage duration, or differences in malignancy characteristics. Further investigation is warranted. Fifth, our analysis did not evaluate all factors that might influence cfDNA quantity and quality, including collection tube types, anticoagulants, processing temperatures, tube agitation, centrifugation conditions, the number of freeze-thaw cycles, extraction methods, and quantification techniques.

## 5. Conclusions

In summary, our study identified key factors affecting cfDNA analysis in long-term stored plasma samples. While cfDNA yield remained largely unchanged, prolonged plasma storage increased gDNA contamination and reduced 5hmC sequencing quality. We recommend complete removal of DNA-containing components from plasma, prompt plasma processing after blood collection, and minimizing plasma storage duration to improve the quality of downstream analysis. This knowledge is crucial for cfDNA biomarker research and for large-scale, cross-center validation studies and clinical trials evaluating the clinical utility of cfDNA and its integration into routine practice.

## Figures and Tables

**Table 1:** Characteristics of patient and control samples.

	Total	Solid Tumor	Bladder	Breast	Colorectal	Kidney	Lung	Ovary	Prostate	Uteri	AML	Control
Sample size	1070	622	45	71	67	63	125	10	227	14	237	211
(male)	(612)	(392)	(27)	(1)	(44)	(40)	(53)	(0)	(227)	(0)	(123)	(97)
Age (years) range	20–95	24–95	37–91	24–91	40–95	35–88	46–92	29–83	40–84	37–86	27–94	20–93
(median)	(65)	(66)	(74)	(54)	(66)	(67)	(70)	(63)	(63)	(70.5)	(63)	(60)
Male	20–95	35–95	37–91	82–82	46–95	35–88	53–92	NA	40–84	NA	31–88	20–90
	(65)	(66)	(76)	(82)	(65)	(67)	(70)		(63)		(66)	(64)
Female	21–94	24–91	51–88	24–91	40–89	49–82	46–90	29–83	NA	37–86	27–94	21–93
	(63)	(65)	(71.5)	(53.5)	(68)	(65)	(70)	(63)		(70.5)	(61.5)	(59)
Stage 0	18	18	10	7	1	0	0	0	0	0	NA	NA
Stage I	178	178	12	30	11	39	32	1	49	4	NA	NA
Stage II	190	190	7	11	6	11	11	5	154	1	NA	NA
Stage III	61	61	3	7	8	11	11	5	15	1	NA	NA
Stage IV	87	87	8	2	5	3	63	3	2	1	NA	NA
Stage NA	536	88	5	14	36	7	11	1	7	7	237	211
Plasma storage years	≤14	≤7	1–6	2–7	4–7	2–7	≤7	2–4	1–6	1–6	≤14	≤13
(median)	(5.3)	(5.1)	(5.1)	(5.3)	(5.7)	(5.9)	(0.4)	(3.5)	(5)	(3.7)	(1.3)	(10.7)
Elapsed days	0–36	0–36	0–9	0–5	0–0	0–1	0–36	1–6	0–30	1–8	0–27	0–4
(median)	(1)	(2)	(2)	(1)	(0)	(0)	(1)	(1.5)	(2)	(3)	(1)	(1.5)
Sample size with elapsed time	643	415	43	6	2	5	112	10	223	14	220	8

**Table 2:** Multivariable quantile regression analysis of cfDNA yield.

	*p* Value (Coefficient)		
Quantile	25th	50th	75th
AML			
Age	0.14 (0.21)	0.02 ^#^ (0.94)	0.76 (0.43)
Sex	0.97 (0.15)	0.38 (−11.14)	0.13 (−73.60)
Storage time	0.35 (1.35)	0.008 ^#^ (5.58)	0.39 (4.50)
gDNA contamination	0.04 ^#^ (9.70)	0.48 (−8.2)	0.96 (1.85)
Elapsed days	0.74 (−0.67)	0.58 (−2.56)	0.72 (4.34)
Solid Tumor			
Age	0.53 (0.03)	0.15 (0.13)	0.28 (−0.29)
Sex	0.40 (1.90)	0.12 (4.75)	0.13 (9.52)
Storage time	0.79 (−0.11)	0.80 (−0.18)	0.42 (−1.08)
gDNA contamination	0.07 (2.51)	0.07 (4.07)	0.83 (0.96)
Stage IV	0.73 (1.64)	0.17 (10.03)	0.48 (14.50)
Elapsed days	0.44 (−0.13)	0.93 (0.008)	0.12 (1.51)
Control			
Age	0.07 (0.12)	0.48 (0.09)	0.60 (−0.18)
Sex	0.26 (−3.24)	0.33 (−5.90)	0.26 (−12.56)
Storage time	0.34 (−0.38)	0.51 (−0.53)	0.34 (1.50)
gDNA contamination	0.28 (−3.86)	0.21 (−9.01)	0.16 (−23.89)

^#^ < 0.05.

**Table 3:** Multivariable logistic regression analysis of cfDNA fragment presence, gDNA contamination, and success of 5hmC library preparation.

	*p* Value (Coefficient)		
	cfDNA Fragment Presence	gDNA Contamination	Successful DNA Library Preparation
AML			
Age	0.96 (8.6 × 10^−4^)	0.15 (0.01)	0.07 (−0.03)
Sex	0.69 (−0.17)	0.52 (0.18)	0.76 (0.15)
Storage time	0.97 (−0.002)	0.05 ^#^ (0.06)	0.19 (0.10)
cfDNA Yield	-	-	4.1 × 10^−4^ ^#^ (0.05)
gDNA contamination	0.33 (0.43)	-	0.18 (0.64)
Elapsed days	0.29 (−0.06)	0.94 (−0.004)	0.26 (0.24)
Solid Tumor			
Age	0.15 (0.02)	0.02 ^#^ (−0.03)	0.24 (0.02)
Sex	0.41 (0.36)	0.007 ^#^ (−0.77)	8.6 × 10^−4^ ^#^ (2.49)
Storage time	0.08 (−0.17)	4.9 × 10^−4^ ^#^ (0.23)	0.49 (0.07)
cfDNA Yield	-	-	0.004 ^#^ (0.04)
gDNA contamination	0.01 ^#^ (−0.81)	-	0.008 ^#^ (−0.99)
Stage I	0.90 (0.11)	0.39 (−0.63)	0.99 (−14.57)
Stage II	0.75 (−0.26)	1.0 (−6.9 × 10^−4^)	0.98 (−15.43)
Stage III	0.50 (0.69)	0.33 (−0.77)	0.98 (−15.45)
Stage IV	0.57 (0.56)	0.42 (−0.64)	0.99 (−13.05)
Elapsed days	0.70 (−0.01)	0.004 ^#^ (0.15)	0.84 (0.009)
Control			
Age	0.09 (0.02)	0.27 (−0.01)	0.11 (0.02)
Sex	0.19 (−0.54)	0.05 ^#^ (−0.66)	0.25 (−0.53)
Storage time	0.51 (0.05)	0.92 (0.006)	0.92 (0.007)
cfDNA Yield	-	-	0.02 ^#^ (0.04)
gDNA contamination	0.99 (−18.21)	-	0.30 (−0.58)

^#^ < 0.05.

**Table 4:** Multivariable quantile regression analysis of uniquely mapped reads.

	*p* Value (Coefficient)		
Quantile	25th	50th	75th
AML			
Age	0.58 (13,759.06)	0.50 (−27,088.05)	0.63 (18,415.88)
Sex	0.29 (−788,370.44)	0.03 ^#^ (−2,096,216.92)	0.15 (−1,466,351.94)
Storage time	0.02 ^#^ (187,012.66)	0.35 (108,174.20)	0.33 (117,107.39)
cfDNA Yield	0.01 ^#^ (2853.62)	0.34 (1807.65)	0.12 (3090.76)
gDNA contamination	0.15 (−1,429,295.73)	8.6 × 10^−4^ ^#^ (−2,992,290.16)	0.006 ^#^ (−3,297,202.95)
Elapsed days	0.98 (−6300.18)	0.22 (295,514.60)	0.38 (185,534.03)
Solid Tumor			
Age	0.91 (4456.51)	0.27 (32,412.75)	0.38 (43,104.00)
Sex	0.01 ^#^ (2,723,525.42)	0.01 ^#^ (2,658,007.93)	0.005 ^#^ (3,424,488.43)
Storage time	0.71 (−79,012.44)	0.62 (−101,407.76)	0.29 (−279,531.37)
cfDNA Yield	0.46 (−7055.65)	0.97 (363.78)	0.88 (2039.74)
gDNA contamination	0.008 ^#^ (−1,817,579.10)	0.001 ^#^ (−3,147,950.68)	0.01 ^#^ (−3,084,351.72)
Stage I	0.13 (−1,972,118.07)	0.26 (−2,262,700.55)	0.54 (1,320,602.73)
Stage II	0.07 (−2,346,599.28)	0.009 ^#^ (−4,603,701.28)	0.09 (−3,241,460.25)
Stage III	0.57 (−962,704.24)	0.17 (−2,825,400.68)	0.66 (−1,139,338.78)
Stage IV	0.96 (−74,919.75)	0.93 (−174,490.45)	0.22 (2,792,144.28)
Elapsed days	0.35 (94,737.29)	0.50 (81,313.49)	0.15 (221,878.45)
Control			
Age	0.71 (−10,035.71)	0.82 (−6904.97)	0.31 (−32,792.94)
Sex	0.95 (58,911.68)	0.73 (312,244.64)	0.71 (535,284.47)
Storage time	0.003 ^#^ (−827,786.97)	1.5 × 10^−9^^#^ (−1,274,748.90)	5.6 × 10^−10^^#^ (−1,283,080.89)
cfDNA Yield	0.004 ^#^ (20,045.15)	0.03 ^#^ (15,720.95)	0.43 (9131.48)
gDNA contamination	0.18 (−1,436,569.53)	0.19 (−1,320,681.69)	0.04 ^#^ (−3,128,848.41)

^#^ < 0.05.

## Data Availability

The raw 5hmC sequencing data are available in the National Center for Biotechnology Information Gene Expression Omnibus database (https://www.ncbi.nlm.nih.gov/geo/query/acc.cgi?acc=GSE202988, accessed on 23 September 2022. https://www.ncbi.nlm.nih.gov/geo/query/acc.cgi?acc=GSE163846, accessed on 15 June 2022. https://www.ncbi.nlm.nih.gov/geo/query/acc.cgi, accessed on 28 February 2024).

## Supplementary Materials

The following supporting information can be downloaded at: https://www.mdpi.com/article/10.3390/biom15070927/s1, Figure S1: Multivariable quantile regression analysis of cell-free DNA (cfDNA) yield; Figure S2: Evaluation of cfDNA fragment integrity; Figure S3: Evaluation of gDNA contamination; Figure S4: Analysis of 5hmC library preparation success; Figure S5: Multivariable quantile regression analysis of unique mapping reads; Table S1: Correlation of elapsed time and gDNA contamination in solid tumors.
