# Supplementary material for: Impact of Long-Term Plasma Storage on Cell-Free DNA Epigenetic Biomarker Studies
Source: Biomolecules. 2025 Jun 25;15(7):927. doi: 10.3390/biom15070927 (PMC12292636; doi:10.3390/biom15070927)
Supplement: Supplementary file 1 [file biomolecules-15-00927-s001.zip › biomolecules-3685614-supplementary.pdf]

Supplementary

Figure S1. Multivariable quantile regression analysis of cell-free DNA (cfDNA) yield.

Figure S2. Evaluation of cfDNA fragment integrity.

Figure S3. Evaluation of gDNA contamination.

Figure S4. Analysis of 5hmC library preparation success.

Figure S5. Multivariable quantile regression analysis of unique mapping reads.

Table S1. Correlation of elapsed time and gDNA contamination in solid tumors.

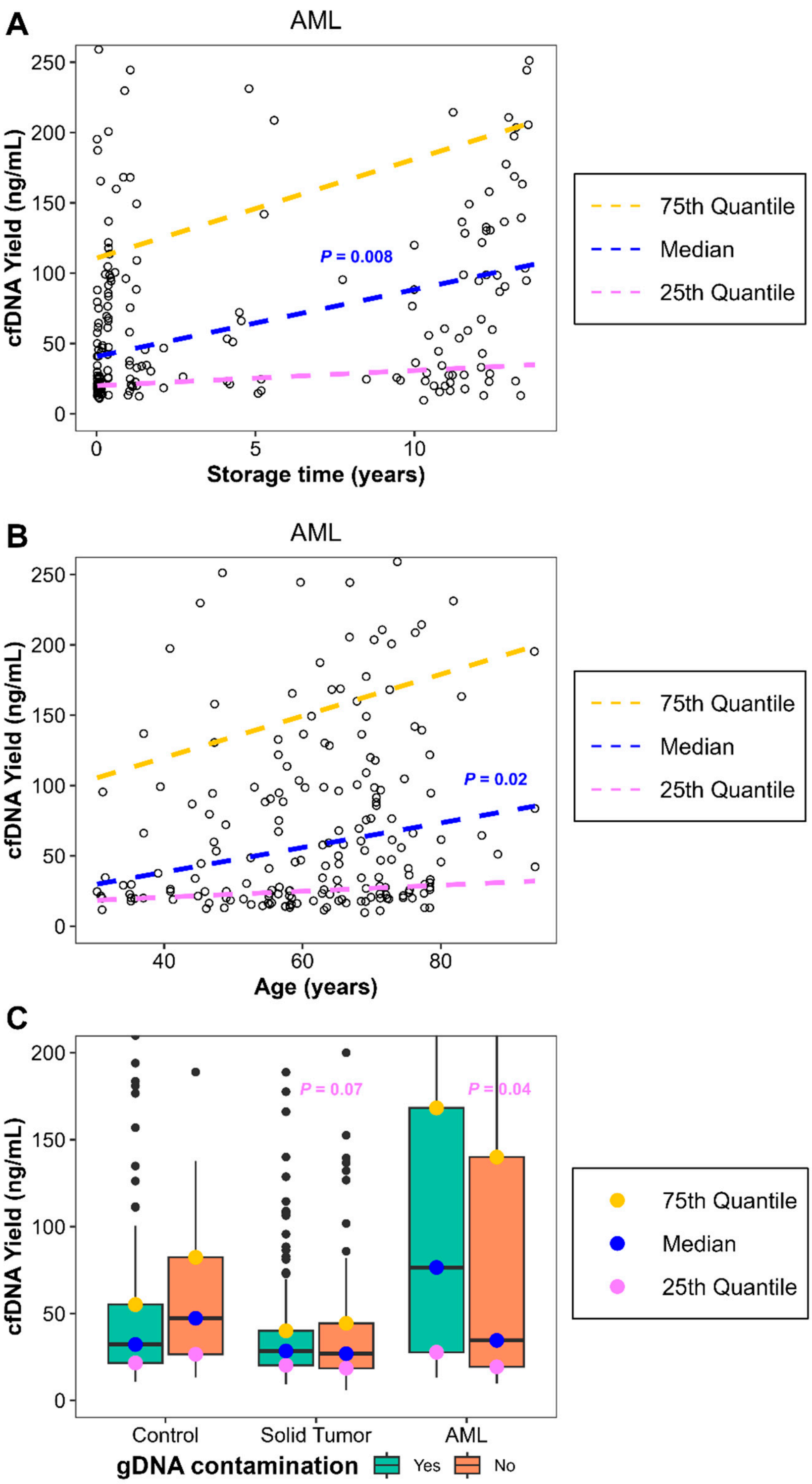

Figure S1. Multivariable quantile regression analysis of cell-free DNA (cfDNA) yield based on plasma storage time (A), age (B), and genomic DNA (gDNA) contamination (C). Multivariable quantile regression analysis was performed at the 25th, 50th, and 75th quantiles of the cfDNA yield. *P* values < .05 are indicated.

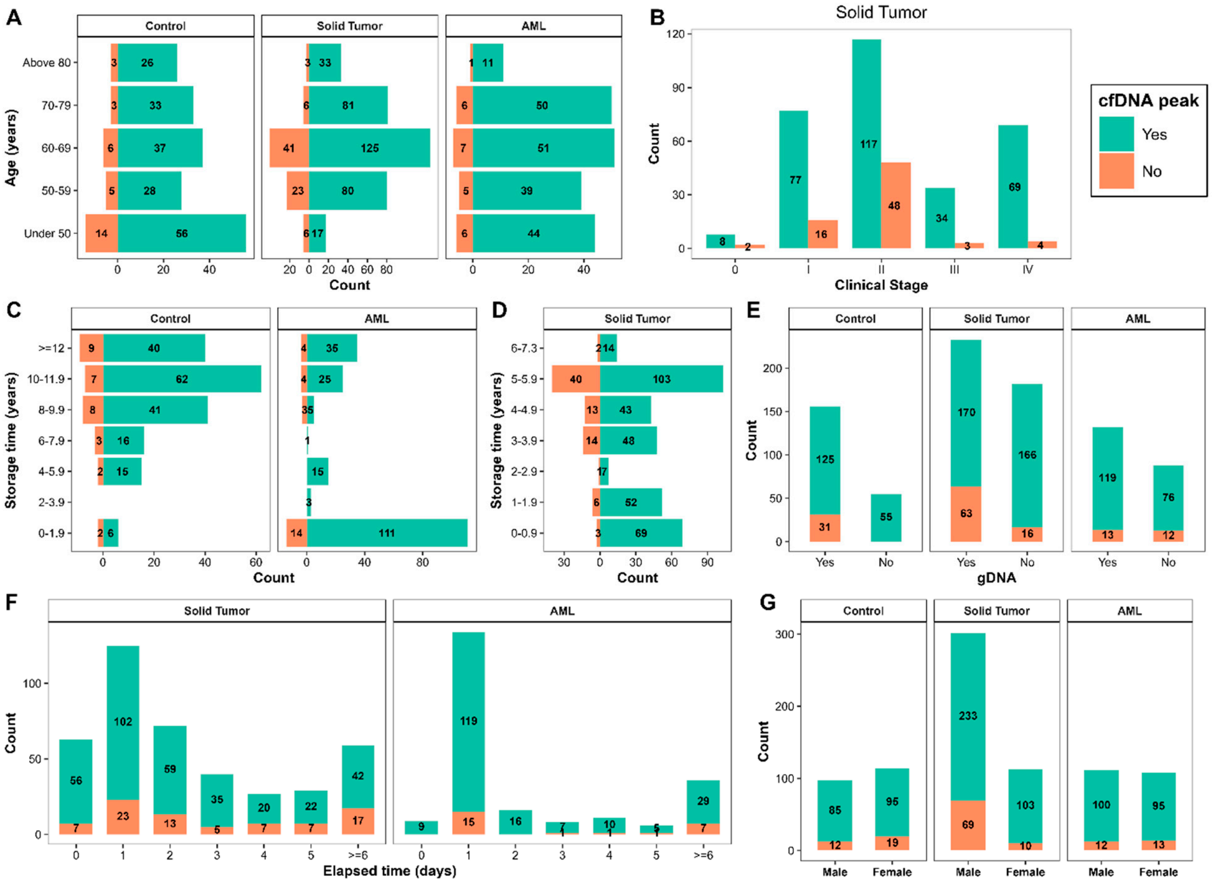

Figure S2. Evaluation of cfDNA fragment integrity. (A–G) Plasma sample number distribution in those with or without cfDNA fragments based on age (A), cancer clinical stage in solid tumors (B), plasma storage time in control and AML (C), plasma storage time in solid tumor (D), gDNA contamination (E), elapsed processing time (F), and sex (G).

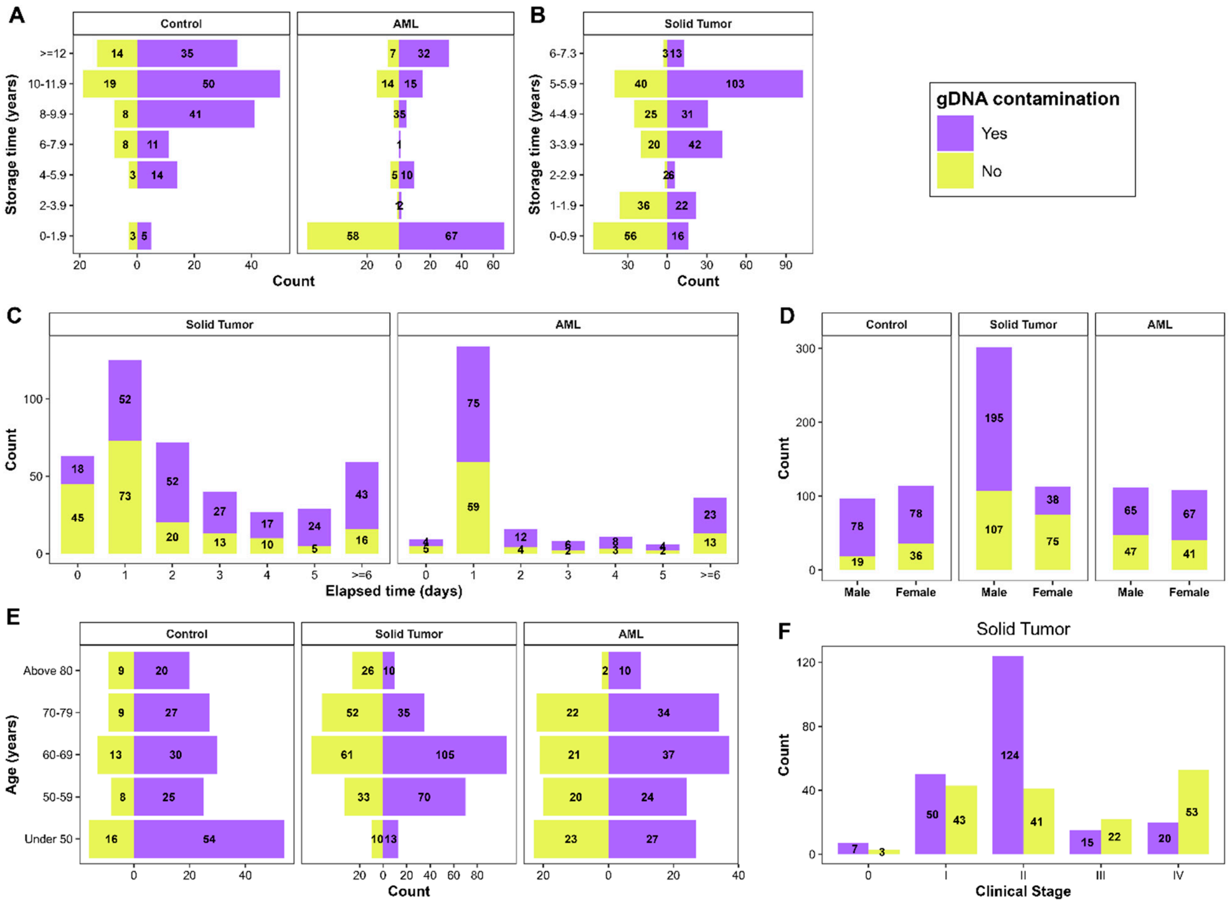

Figure S3. Evaluation of gDNA contamination. (A–F) Plasma sample number distribution in samples with or without gDNA contamination based on plasma storage time in control and AML (A), plasma storage time in solid tumor (B), elapsed time (C), sex (D), age (E), cancer clinical stage in solid tumors (F).

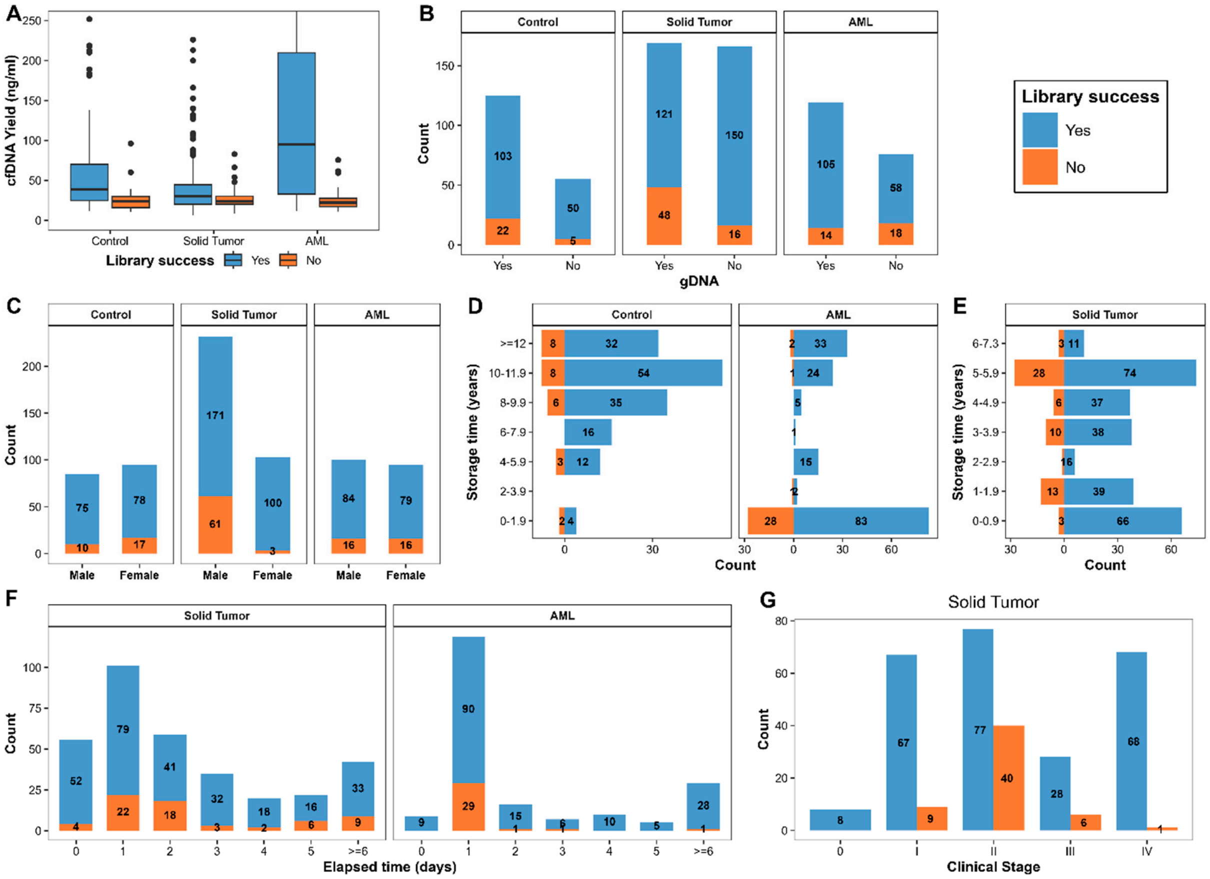

Figure S4. Analysis of 5hmC library preparation success. (A) cfDNA yield in samples with or without 5hmC library preparation success. (B–F) Plasma sample number distribution based on gDNA contamination (B), sex (C), plasma storage time in control and AML (D), plasma storage time in solid tumor (E), elapsed time (F), and cancer clinical stage in solid tumors (G).

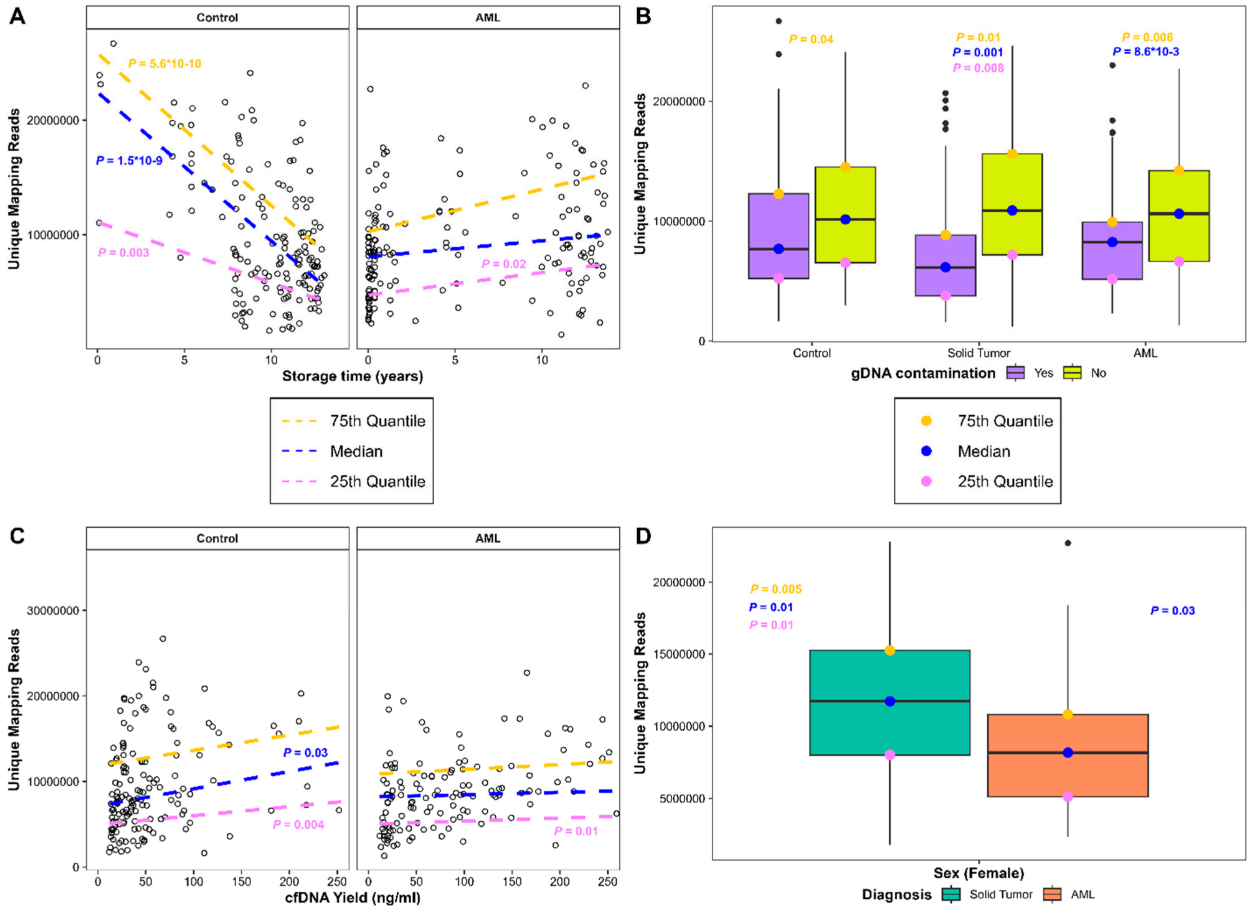

Figure S5. Multivariable quantile regression analysis of unique mapped reads (UMRs) based on plasma storage time (A), gDNA contamination (B), cfDNA yield (C), and sex (D). Multivariable quantile regression analysis was performed at the 25th, 50th, and 75th quantiles of the UMRs. *P* values < .05 are indicated.

Table S1. Correlation of elapsed time and gDNA contamination in solid tumors.

	***P* value (Coefficient)**[1]
**Day 1**	0.08 (0.58)
**Day 2**	1.1*10-6# (1.87)
**Day 3**	1.7*10-4# (1.65)
**Day 4**	0.003# (1.45)
**Day 5**	1.1*10-5# (2.48)
**Day ≥6**	2.5*10-6# (1.90)

[1] Logistic Regression Analysis

< 0.05
